# Mapping burned areas in Thailand using Sentinel-2 imagery and OBIA techniques

**DOI:** 10.1038/s41598-024-60512-w

**Published:** 2024-04-26

**Authors:** Chanida Suwanprasit, Shahnawaz

**Affiliations:** 1 Department of Geography, Faculty of Social Sciences, Chiang Mai University, Chiang Mai, Thailand; 2 Department of Geoinformatics - Z_GIS, University of Salzburg, Salzburg, Austria

**Keywords:** Burned area mapping, Sentinel-2 imagery, Object-based image analysis (OBIA), Thailand, Natural hazards, Fire ecology

## Abstract

Monitoring burned areas in Thailand and other tropical countries during the post-harvest season is becoming increasingly important. High-resolution remote sensing data from Sentinel-2 satellites, which have a short revisit time, is ideal for accurately and efficiently mapping burned regions. However, automating the mapping of agriculture residual on a national scale is challenging due to the volume of information and level of detail involved. In this study, a Sentinel-2A Level-1C Multispectral Instrument image (MSI) from February 27, 2018 was combined with object-based image analysis (OBIA) algorithms to identify burned areas in Mae Chaem, Chom Thong, Hod, Mae Sariang, and Mae La Noi Districts in Chiang Mai, Thailand. OBIA techniques were used to classify forest, agricultural, water bodies, newly burned, and old burned regions. The segmentation scale parameter value of 50 was obtained using only the original Sentinel-2A band in red, green, blue, near infrared (NIR), and Normalized Difference Vegetation Index (NDVI). The accuracy of the produced maps was assessed using an existing burned area dataset, and the burned area identified through OBIA was found to be 85.2% accurate compared to 500 random burned points from the dataset. These results suggest that the combination of OBIA and Sentinel-2A with a 10 m spatial resolution is very effective and promising for the process of burned area mapping.

## Introduction

Chiang Mai has become more polluted due to haze severe and increasing during the dry season every year. Various factors caused haze pollution in Chiang Mai, from urbanization and vehicles exhaust to burning dry leaves and the increasing forest fires and open burning on corn/maize farms, which expanded rapidly due to the demand for corn/maize^1^. The highest particulate matter with an effective aerodynamic diameter smaller than 10 µm (PM10) value in Chiang Mai Province, Thailand, was found in February and continuously decreased in March, April, and May, respectively^2^. After harvesting crops, what remains in an agricultural area is known as “crop residue.” Typically, it can either be plowed directly into the ground or burned before being incorporated into the soil. Besides causing pollution in the air, it is now also a significant generator of greenhouse gases. In many countries, farmers use crop residue burning to quickly dispose of enormous volumes of agricultural waste at the end of each harvest season. The main commonalities across countries in this region are the burning of agricultural leftovers and seasonal forest fires. Many rice-growing regions such as the Philippines, China, India, Vietnam, and Thailand often burn rice husks. These husks can play an important role in local and regional air pollution and worldwide climate change^3^.

The mapping of burned areas has been essential in remote sensing research. Many studies have attempted to quantify and map crop residue burning areas for calculating greenhouse gas emissions using indirect methods such as satellite imagery; optical satellite data have been used extensively for many years in the quantifying and detecting of burned areas^4–9^. The Sentinel-2 mission, a collaborative effort between the European Space Agency (ESA) and the European Commission (EC), has been offering free optical imagery with high spatial resolution (10–20 m) and frequent updates (every ten days, or five days with the combined constellation) since its inception in 2015. To put up a nationwide burned area mapping service, Sentinel-2 data provide high spatial resolution (10–20 m, depending on which band), rich spectral information (more or less a superset of Landsat Enhanced Thematic Mapper Plus (ETM+) and Landsat 8 Operational Land Imager (OLI) bands, and a five-day temporal frequency), making them ideal. The use of Sentinel-2 data for burned area mapping has been investigated in only a few studies^10–15^, and even fewer studies have suggested automating mapping workflows^16–18^.

Image analysis in remote sensing has conventionally utilized techniques such as principal component analysis (PCA), spectral mixture analysis, logistic regression modelling, supervised classification, multi-temporal image compositing algorithms, and spectral indices thresholding. Recently, object-based image analysis (OBIA) has garnered increasing attention across various remote sensing applications. This approach shifts the focus from the traditional “pixel in space” paradigm to an “object in space” perspective^19^. In OBIA, images are segmented based on predefined criteria of homogeneity and heterogeneity, leading to the formation of distinct objects. Each segmented object is then characterized by its spectral, textural, morphological, and contextual attributes. This method effectively reduces the ‘salt-and-pepper’ noise inherent in pixel-based categorization by assigning a single class to the entire object.

Polychronaki and Gitas^20^ demonstrated the high accuracy of OBIA in detecting burned areas using SPOT-4 High-Resolution Visible and Infrared Sensor (HRVIR) images. Furthermore, a recent study introduced an automated framework that integrates machine learning with Sentinel-2 satellite imagery and OBIA for rapid assessment of wildfire damage. This novel approach, which employs training samples derived from textural changes and the Normalized Difference Vegetation Index (NDVI), effectively classifies areas as burned or unburned. When applied to wildfires in South Korea, this methodology achieved impressive overall accuracies of 97.6% in Uljin and 93.8% in Gangneung, underscoring its efficacy and precision for prompt wildfire response and mitigation^21^. Another significant application of OBIA was in assessing damage from a major forest fire on the Spanish Mediterranean coast, utilizing the NOAA-AVHRR sensor. The resultant map of the burned area demonstrated approximately 90% spatial concordance with the data provided by the Catalan Environmental Department, further evidencing the utility of OBIA in large-scale fire damage assessment^22^.

This study utilizes Sentinel-2 MSI imagery, capitalizing on its 10 m spatial resolution in specific spectral bands, for precise mapping of burned areas. The NDVI, derived from red and NIR bands, is key in distinguishing between burned and unburned areas, enabling detailed analysis of burn scars. The objective is to apply OBIA techniques with Sentinel-2 imagery for identifying burned regions in Ban Thap subdistrict, Mae Chaem District, Chiang Mai, Thailand. Although focused on Chiang Mai, the research has wider relevance. The methods used here can be adapted for similar environmental issues in other regions. This article aims to elucidate how Sentinel-2 data and OBIA can be effectively used for environmental monitoring and management. The innovative combination of OBIA and Sentinel-2’s detailed imagery provides a robust framework for accurate mapping and assessment of areas impacted by agricultural burning and forest fires. This not only enhances understanding of the environmental effects in Chiang Mai but also suggests applications for these techniques in diverse geographical areas facing comparable challenges. Therefore, this study's insights and methodologies have global significance, especially in regions where agricultural and forest fire practices significantly affect the environment and public health.

## Materials and methods

### Remote sensing of burned area

Following forest fires or land burning events, charcoal residue and vegetation scars are key in remote sensing for detecting and assessing burned areas. Charcoal residue, a byproduct of combustion, exhibits a unique spectral signature, absorbing visibly and reflecting in the infrared range, making it easily detectable in satellite imagery for persistent remote sensing analysis. Vegetation scars, marking fire-affected areas, undergo physical and chemical changes, such as color and texture alterations due to chlorophyll loss and stem charring, resulting in less red light absorption and more NIR reflection, thereby serving as reliable burned area indicators. These features define the post-fire landscape, offering extended spectral signals crucial for evaluating fire damage's ecological and economic impact. Burned lands are characterized by carbon and ash deposition, plant extinction, and altered vegetation structure, with white mineral ash production increasing NIR and visible reflectance. The loss of the plant layer reduces canopy moisture and shadow, typically radiation absorbers. Thermal infrared radiation (TIR) becomes effective for smoke detection as fire incidents elevate temperatures, and short wave infrared radiation (SWIR) detects water stress in vegetation and the presence of burned vegetation, indicating temperature increases during fires. These spectral changes and characteristics are integral to identifying and continuously monitoring burned regions through remote sensing techniques.

#### Normalized difference vegetation index (NDVI)

NDVI is instrumental in distinguishing vegetation health and coverage. It is widely used for assessing vegetation health and vigor and can be influenced by various environmental factors such as atmospheric conditions, which can lead to potential inaccuracies in burned area mapping. However, its primary benefit lies in its ability to clearly differentiate between healthy vegetation and burned areas, as healthy vegetation exhibits higher NDVI values due to its greater reflectance in the NIR spectrum. This makes NDVI particularly useful in quickly identifying and assessing the extent of burned areas, especially in regions with dense vegetation^23^. Normally, high NDVI values, approaching +1, indicate dense and healthy vegetation, characterized by strong absorption of visible red light by chlorophyll and high reflection in the NIR spectrum. Conversely, low NDVI values, close to 0 or negative, suggest sparse, unhealthy vegetation or non-vegetative surfaces such as bare soil, water, or burned areas. NDVI is one of the most popular tools for detecting burned or damaged vegetation due to its ease of use and generally excellent performance^24^. When a plant is burned or otherwise damaged, there is often a significant decrease in its NDVI value, making it easier to identify burn or damage areas. NDVI is commonly used to map burned regions^6,23^, quantify burn severity^12,26,27^, and monitor vegetation recovery^28,29^. This is because NDVI is sensitive to changes in the chlorophyll content of leaves, which is typically reduced in burned or damaged vegetation. In the context of burned area mapping, this index is particularly valuable; post-fire, NDVI values drop significantly due to vegetation loss, making it a reliable metric to map and assess the extent of burned areas.

#### Normalized burn ratio (NBR)

NBR is a common index used to detect and map burned areas in remote sensing images. It is calculated by comparing the reflectance of SWIR and NIR bands, which are sensitive to the presence of vegetation. However, there are limitations to using NBR to detect and map burned areas. It may not always be able to distinguish between burned and unburned vegetation, particularly in cases where a fire occurred during the dry season and there is a lack of green vegetation. In these cases, other techniques such as TIR imaging may be more effective at detecting and mapping burned areas. The accuracy of different spectral bands and indices for detecting and mapping burned areas may also vary based on factors such as vegetation type, regrowth rates, and the timing of the fire^30^. Additionally, the observation of burned areas may be affected by cloud and cloud-shadow interference^31,32^. Another limitation of NBR is that it is sensitive to the age of the burn. NBR values tend to decrease as the burn scar ages, which can make it difficult to accurately map burned areas that occurred in the past. In these cases, other remote sensing techniques such as visible/near-infrared (VNIR) imaging may be more effective at detecting and mapping older burn scars^33^.

#### Other burned indices

In the context of identifying burn areas, various indices like the Simple Ratio Index (SI), Normalized Burn Ratio (NBR), and Normalized Difference Moisture Index (NDMI) are utilized, each offering unique benefits and facing specific limitations^25^. The SI, for instance, is straightforward and effective in highlighting areas of high biomass but may not be as sensitive to subtle vegetation changes or moisture content^34^. NBR is particularly adept at identifying burn severity and delineating burn scars, but its effectiveness can be reduced in regions with low vegetation or in detecting early post-fire regeneration. NDMI is valuable for assessing moisture content in vegetation, which can indirectly indicate fire susceptibility or recovery, yet it might be less effective in dry environments or in differentiating between types of vegetation^35^.

However, NDVI stands out with a distinct advantage. Its ability to detect burned areas in broadleaf plants for up to five years post-fire sets it apart. This longevity in detection capability makes NDVI an invaluable tool for monitoring long-term fire impacts on vegetation. It helps in understanding the recovery process and the resilience of different vegetation types after a fire. This aspect of NDVI is particularly beneficial for ecological studies and land management practices, offering insights into the regenerative dynamics of ecosystems affected by fires. However, similar to other indices, NDVI's effectiveness can be influenced by atmospheric conditions, and its sensitivity might vary across different vegetation types and stages of growth. Despite these limitations, NDVI's long-term monitoring capability enhances its utility in fire-affected ecological assessments^24,35^.

### Study area

**Figure 1:**
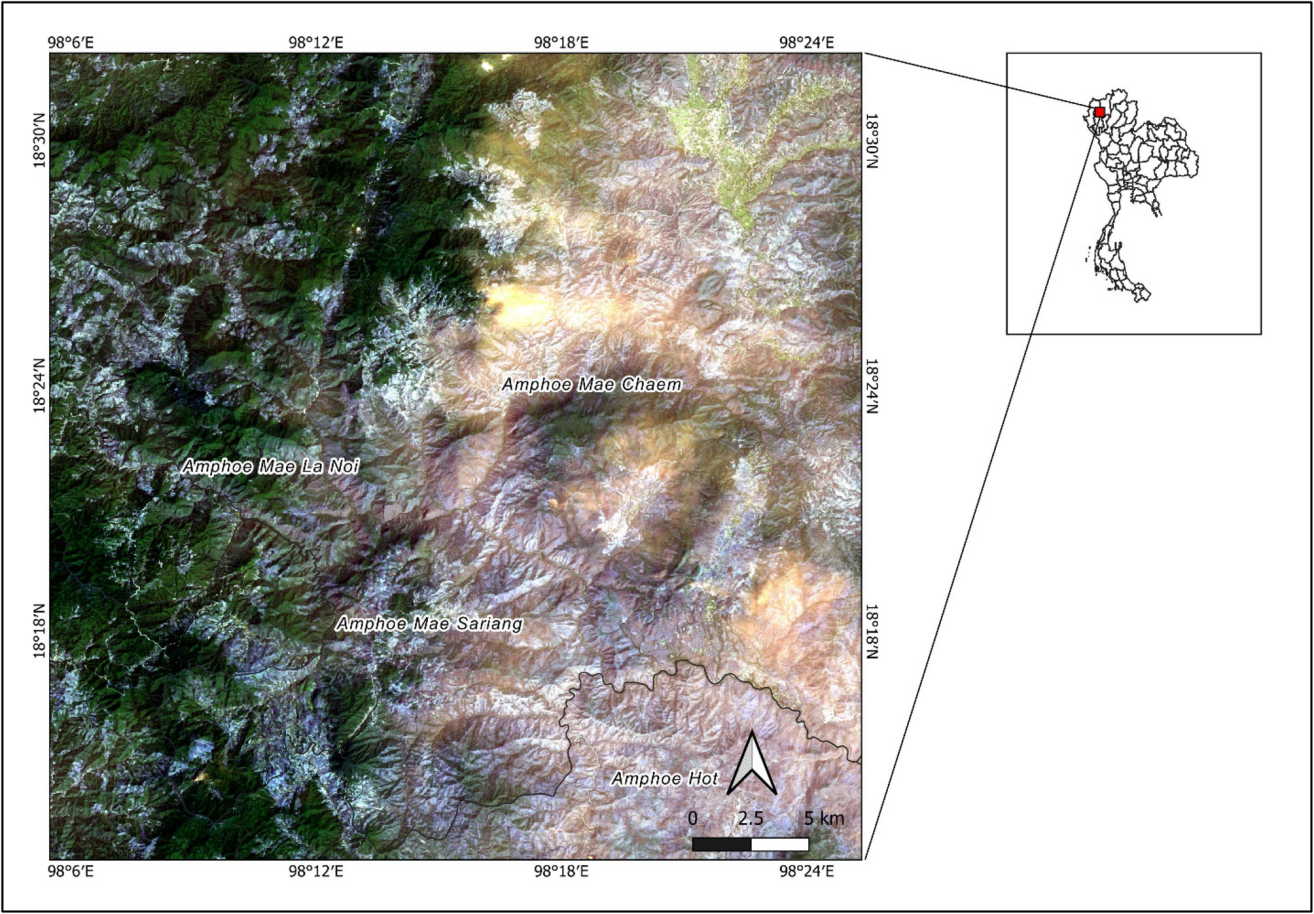
Sentinel-2 true color composite from February 27, 2018, of the study area. (The data for Fig. 1 is sourced from a public Sentinel-2 image available at https://scihub.copernicus.eu/. The processing software used is QGIS version 3.26.2, available at https://qgis.org/en/site/).

The study site is located in Mae Chaem, Chom Thong, Hod, Mae Sariang, and Mae La Noi Districts in Chiang Mai, Thailand (centered at 98.2388, 18.3981) and covers an area of 1766 km^2^ (Fig. 1). According to the 2017 report on burning areas in Thailand by the Geo-Informatics and Space Technology Development Agency (GISTDA), Mae Chaem District had the largest burned area of all the districts, with a total of 370 km^2^. The Ban Thap subdistrict was one of the areas most frequently affected by fires. In the past, shifting cultivation was the most common land use type in this area, but it has recently transitioned to contract farming with crop rotation in monocultures such as maize, rice, and grain^36,37^.

### Data and method

The Sentinel-2A Level-1C MSI image used in this study was acquired by the Sentinel-2 satellite on February 27, 2018. The image was downloaded from the Sentinel Scientific Data Hub, which is a repository of data collected by the Sentinel satellite system. The Level-1C products available on the Scientific Data Hub have been processed by the Payload Data Ground Segment (PDGS) and are radiometrically and geometrically corrected to the top of atmosphere (TOA). This means that the data have been corrected for any distortions caused by the Earth's atmosphere and for any geometric distortions that may have occurred during the data collection process.

The Sentinel-2 satellite is equipped with a MSI that is capable of collecting data in multiple spectral bands, including the red, green, blue, and NIR regions (Table 1). The MSI has a spatial resolution of 10 m, which means that each pixel in the image represents an area of 10 m by 10 m on the ground. In previous studies, Sentinel-2 MSI data has been effectively used to assess burn severity, or the degree to which an area has been affected by a fire. This is because the MSI data is sensitive to changes in the chlorophyll content of vegetation, which is typically reduced in areas that have been burned. In general, the accuracy of burned area mapping increases with higher spatial resolution data. Using a lower spatial resolution image, such as the 20 m spatial resolution of Sentinel-2 data, may result in less accurate burned area maps^10,12,38^.

**Table 1:** Sentinel-2 bands characteristic used in this study.

Sentinel-2 bands	Layer	Central wavelength (µm)	Spatial resolution (m)	Bandwidth (nm)
Band 2—Blue	L1	0.490	10	98
Band 3—Green	L2	0.560	10	45/46 (2A/2B)
Band 4—Red	L3	0.665	10	38/39 (2A/2B)
Band 8—NIR	L4	0.842	10	115

**Figure 2:**
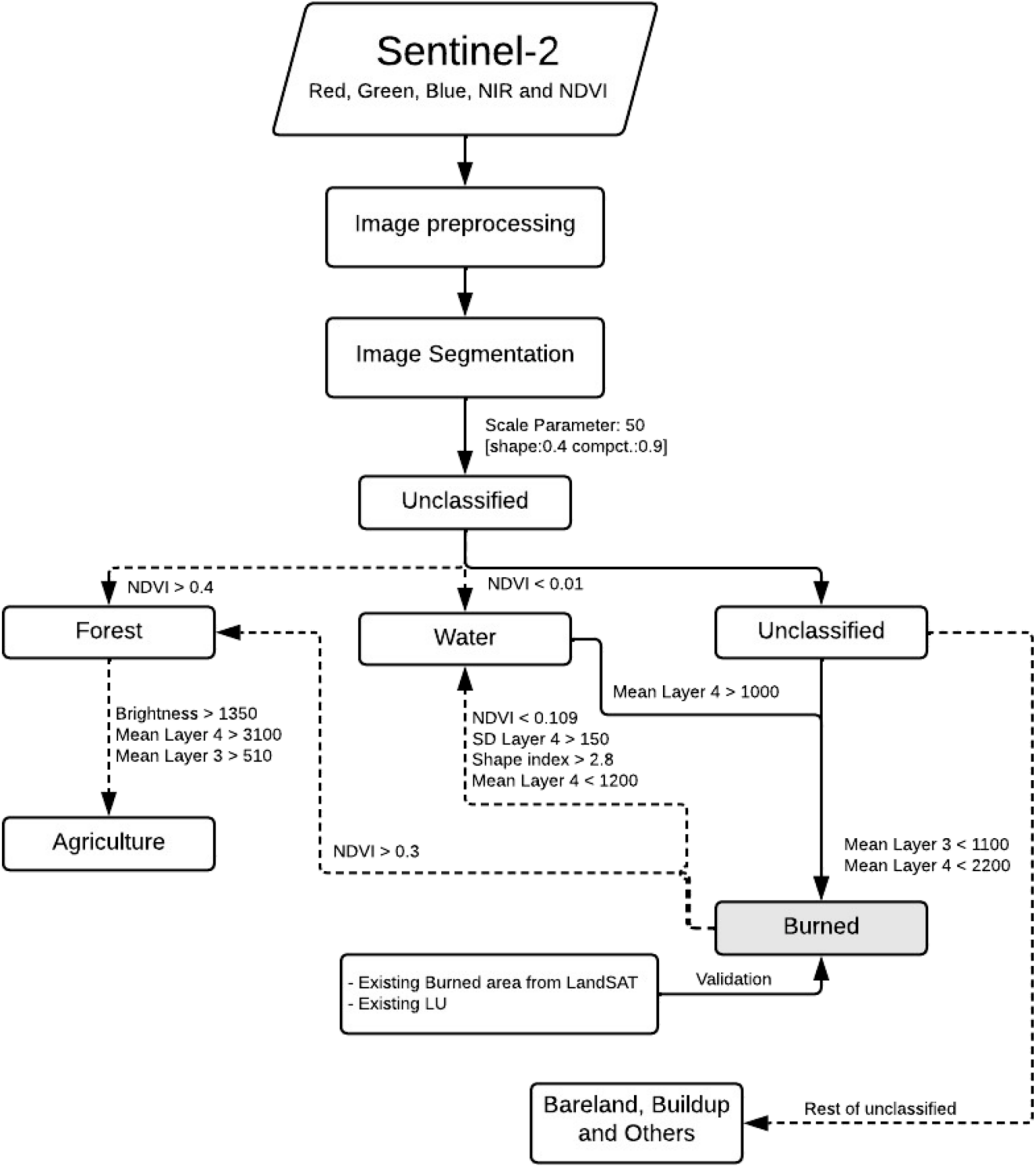
Workflow of OBIA and validation.

The methodology follows a structured workflow (Fig. 2) beginning with image pre-processing to correct for any distortions or noise in the raw satellite data. Following this, image segmentation is conducted using a defined scale parameter and shape index, segmenting the image into distinct objects for more precise classification. Subsequently, the classification process involves using NDVI thresholds and spectral characteristics to categorize the land into various cover types such as forests, water bodies, and burned areas. Additional spectral criteria, such as brightness and mean layer values, are employed to refine the classification between similar land cover types, like distinguishing between forested areas and agricultural land. Finally, the classification results are validated against existing burned area data from Landsat satellites and land use information to ensure the accuracy and reliability of the analysis. This comprehensive approach allows for a detailed assessment of the burned regions, contributing significantly to the field of remote sensing and post-disaster assessment.

#### Pre-processing

In this study, Sentinel-2 data are TOA corrected and geo-referenced. All bands with 10 m spatial resolution (blue, green, red, and NIR) were combined into a single image using band combination for further processing. After that, the NDVI value was determined using Sentinel-2 images. NDVI is a mathematical mixture of the red and NIR bands that yields values ranging from − 1 to + 1, as expressed below (Eq. 1):

1 $$NDVI=\frac{NIR-RED}{NIR+RED}$$

#### Image segmentation

Within the framework of OBIA, Multiresolution Segmentation (MRS) was selected for its adeptness at dividing satellite imagery into meaningful objects that reflect the true heterogeneity of the landscape, an attribute especially advantageous for recognizing subtle variations in burned areas. MRS is a widely used region-based algorithm for land-surface segmentation^39^ which aligns with human visual interpretation, segmenting images in a way that closely resembles the innate variability found in natural environments. It prioritizes homogeneity within these segments, aiding in distinguishing between areas with minor spectral differences. The method also reduces classification errors commonly associated with pixel-based segmentation, leading to more precise and unambiguous results. Crucially, MRS facilitates the incorporation of diverse data layers, such as spectral, textural, and contextual inputs, enhancing the detection of burned areas—a central aspect of this study. Its successful application in previous land cover classifications demonstrates its reliability and reaffirms its selection for this study, aiming to provide a nuanced mapping of post-fire terrain.

MRS operates on a bottom-up algorithmic approach, where pixels are incrementally grouped with their neighbors to form segments until they reach a size defined by a heterogeneity threshold. This threshold is a function of the image layer's weight, scale parameter, and the desired shape and compactness of the segments. The scale parameter dictates the allowable standard deviation for homogeneity within the segments, and together with the shape and compactness criteria, determines the balance between color and form during the segmentation process. This careful calibration ensures that the segmentation results are optimized for the study's specific requirements, yielding a segmented image that accurately represents the diverse characteristics of the landscape post-disturbance.

To obtain appropriate segmentation, the weights, scale parameters, and shapes were experimented differently. The image was segmented using trial and error, with scale values ranging from 50 to 150. All image layers (blue, green, red, NIR, and NDVI) were combined into a single image. After the image was segmented, the objects needed to be classified to identify the burned area objects. The OBIA rule-based classification was developed based on the workflow shown in Fig. 2. The major steps included data preprocessing, NDVI calculation, image segmentation, setting up the ruleset classification, and validating the results.

#### Accuracy assessment

In this study, to assess the accuracy of our burned area maps, a point accuracy assessment method was employed, using the burned area dataset from the GISTDA, a national agency. This dataset, renowned for its 80% accuracy and derived from Landsat 8 imagery corroborated by field surveys and fire hotspot analysis, was chosen for its extensive coverage and the reliability stemming from GISTDA's national authority and expertise in satellite data analysis. Our maps were then compared against GISTDA's data to gauge their accuracy. Despite its advantages, GISTDA's dataset has certain limitations, particularly its sole focus on burned areas without explicit identification of unburned regions, which complicates a comprehensive point accuracy assessment process. Moreover, the lack of detailed accuracy metrics for unburned areas within GISTDA's dataset adds a layer of uncertainty, influencing the precision and reliability of the reference data. This aspect is crucial for the interpretation of our study’s results and in assessing the overall efficacy of our comparative analysis.

Accuracy assessments of OBIA-based burned area mapping have shown promising results, with accuracies typically ranging from 42 to 96%, with an average of 85%^40^. However, it is important to note that the accuracy of OBIA-based burned area mapping can be affected by several factors, such as the quality of the input imagery, the selection of segmentation parameters, and the choice of classification features^40^.

## Results and discussion

**Figure 3:**
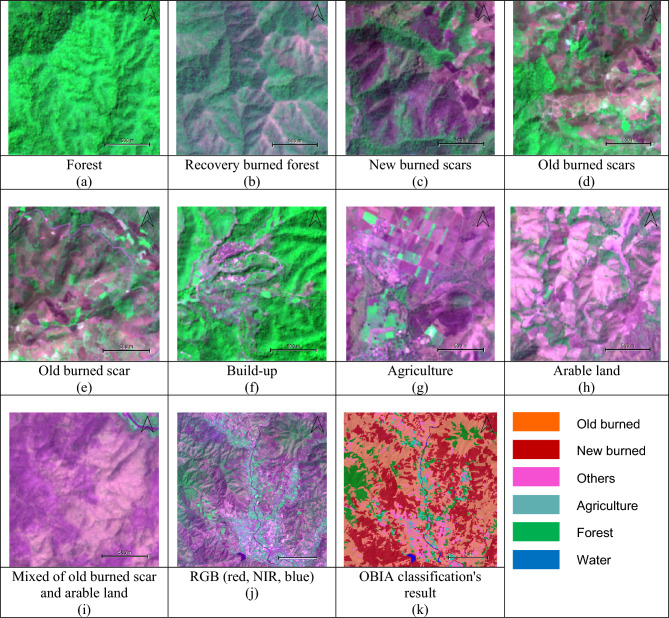
Examples of area characteristics (**a**) Forest, (**b**) Recovery burned forest, (**c**) New burned scars, (**d**) Old burned scars, (**e**) Old burned scar, (**f**) Build-up, (**g**) Agriculture, (**h**) Arable land, and (**i**) mixed of old burned scar and arable land, (**j**) RGB (red, NIR, blue), and (**k**) OBIA classification's result. (This figure is the result of our analysis. The raw data was sourced from a public Sentinel-2 image available at https://scihub.copernicus.eu/. The segmentation analysis was conducted using Definiens eCognition version 10.3, which is available at https://geospatial.trimble.com/. The software was provided by the Department of Geoinformatics – Z_GIS, University of Salzburg, Austria. Further processing was done using QGIS version 3.26.2, available at https://qgis.org/en/site/).

Sentinel-2 multispectral imagery revealed that agricultural areas were the most affected by the fires. To detect the burned regions, this study used false color compositing with the RGB (red, NIR, blue) bands. The resulting image was used to identify different land cover types, including forest, recovery burned forest, newly burned scars, old burned scars, built-up areas, agriculture, arable land, and areas with a mix of old burned scars and arable land. False color compositing can help to highlight burned areas and make them more distinct from other land cover types (Fig. 3).

**Figure 4:**
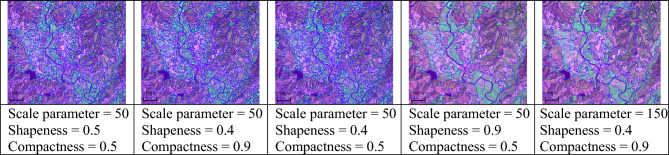
Examples of testing different scale parameters for image segmentation in the study. (This figure is the result of our analysis. The raw data was sourced from a public Sentinel-2 image available at https://scihub.copernicus.eu/. The segmentation analysis was conducted using Definiens eCognition version 10.3, which is available at https://geospatial.trimble.com/. The software was provided by the Department of Geoinformatics – Z_GIS, University of Salzburg, Austria. Further processing was done using QGIS version 3.26.2, available at https://qgis.org/en/site/).

For the image segmentation, attaining precise segmentation necessitates calibrating the scale parameter so the segmentation algorithm optimally delineates individual objects. Through a methodical approach, varying scale parameters from 50 to 150 were trialled to identify a value that neither oversimplifies the image into broad, indistinct areas nor divides it into an overly complex array of minute segments. The scale parameter of 50 emerged as the ideal, facilitating accurate boundary detection while avoiding the pitfalls of merging unique objects into indistinguishable clusters or, alternatively, dissecting the image into an overly detailed patchwork. This carefully chosen scale parameter is depicted in Fig. 4, which illustrates a well-balanced segmentation that captures the true heterogeneity and spatial arrangement of the image features.

Figure 4 also exhibits the results of fine-tuning not only the scale but also the shape and compactness parameters, highlighting the importance of their interrelated adjustments. A higher scale parameter can lead to uniform segments that merge distinct entities, erasing finer details essential for individual object recognition. In contrast, a lower scale parameter increases sensitivity to minute differences, resulting in a granulated segmentation that may overwhelm the classification and analysis process. The experimental outcomes, as displayed, emphasize that a chosen scale of 50, in conjunction with optimal shape and compactness values, is critical to achieve a segmentation that faithfully represents the objects' spatial distribution within the image.

**Figure 5:**
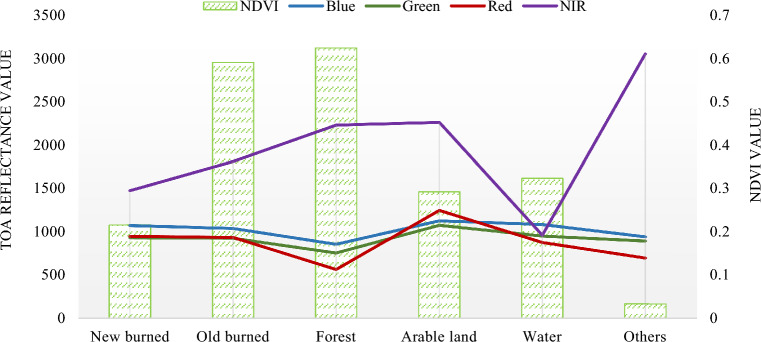
Average band value in each land cover type.

Figure 5 shows the average spectral response of the blue, green, and red bands for new and old burned areas and forests. The spectral response of the new burned area is slightly higher in these bands than the old burned area, except for the NIR band. The NDVI value of the old burned area is also lower than the new burned area and the forest area. This graphical representation is instrumental in elucidating the spectral differences between burned and unburned forest areas. Specifically, new burned areas exhibit a unique spectral signature, particularly in the NIR band, with lower NDVI values indicating significant vegetation damage or loss. In contrast, forest areas display a spectral profile distinct from burned areas, especially in the NIR and red bands, reflecting the characteristics of healthy, chlorophyll-rich vegetation.

Complementing this, Table 2 shows the minimum and maximum value ranges for different spectral bands and land cover types. The spectral bands are blue, green, red, NIR, and NDVI. The land cover types are new burned, forest, others, arable land, old burned, and water. In the spectral response analysis conducted, discernible variations were observed across different land cover types, with data derived from zonal statistics of all classification categories. New burned areas are typified by increased spectral values in most bands, excluding the NDVI. This spectral response is indicative of the altered surface characteristics following a burn event, such as the reduction in vegetation cover and the exposure of soil or charred material, leading to enhanced reflectance in the visible and NIR spectrums.

**Table 2:** The minimum and maximum value of spectral response values.

Classification types	Minimum and maximum value range				
	Blue	Green	Red	NIR	NDVI
New burned	743–1763	539–1951	363–2084	469–3235	− 0.187 to 0.638
Forest	660–1603	466–1522	276–1873	613–4511	0.004–0.832
Others	707–2591	626–1580	365–2026	1341–5174	0.139–0.833
Arable land	774–4392	660–5224	455–5844	1183–6673	− 0.054 to 0.713
Old burned	712–2293	528–2360	337–2519	614–4032	− 0.172 to 0.734
Water	810–3158	683–3638	517–4378	556–4355	− 0.257 to 0.475

Conversely, forest areas exhibit lower spectral responses compared to new burn areas, yet higher than other land cover types, a pattern attributable to the dense presence of healthy, chlorophyll-rich vegetation that absorbs more in the red spectrum and reflects more in the NIR spectrum. Older burned areas, as per the zonal statistics, show spectral values lower than those of new burned areas but higher than water bodies, indicating some degree of ecological recovery or regrowth, thus altering their spectral signatures. Water bodies, analyzed through the same zonal statistical approach, consistently demonstrate the most distinct spectral profile with the lowest values in all bands, except the NDVI, due to their inherent spectral properties characterized by substantial absorption and minimal reflectance in the visible and NIR wavelengths. The study found that new burned areas have the highest values in all spectral bands except for NDVI. Forest areas have lower values in all spectral bands than new burned areas, but higher values than other land cover types. Others refer to a mix of different land cover types, so it has a wide range of values in all spectral bands. Arable land has the highest values in the NIR band. Old burned areas have lower values in all spectral bands than new burned areas, but higher values than water. Water has the lowest values in all spectral bands except for the NDVI. The NDVI values are all negative for water, which is due to the high reflectance of water in the NIR band. The NDVI values for other land cover types are all positive, with new burned areas having the lowest values and forest having the highest values.

**Figure 6:**
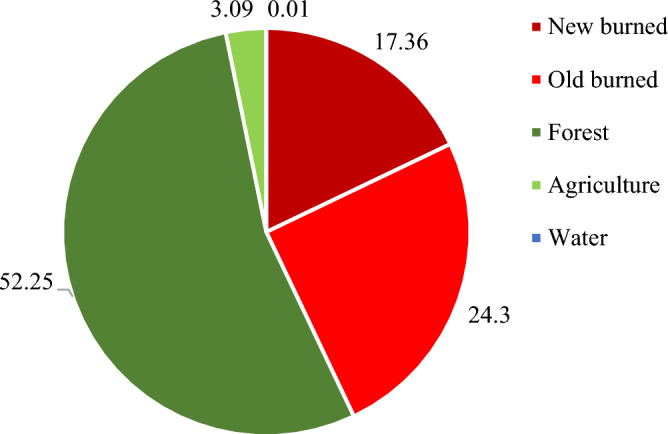
Percentage of classification area from OBIA.

**Figure 7:**
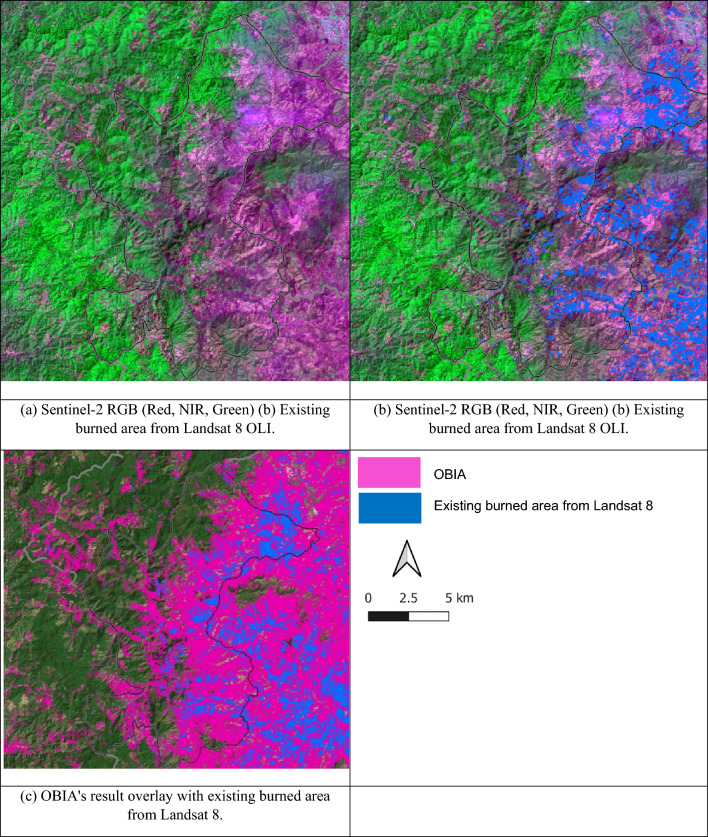
Comparison of (**a**) Sentinel-2 RGB (Red, NIR, Green) (**b**) the existing burned area from Landsat 8 OLI and (**c**) OBIA's result overlay with existing burned area from Landsat 8. (This figure is the result of our analysis. The raw data was sourced from a public Sentinel-2 image available at https://scihub.copernicus.eu/. The segmentation analysis was conducted using Definiens eCognition version 10.3, which is available at https://geospatial.trimble.com/. The software was provided by the Department of Geoinformatics – Z_GIS, University of Salzburg, Austria. Further processing was done using QGIS version 3.26.2, available at https://qgis.org/en/site/).

**Figure 8:**
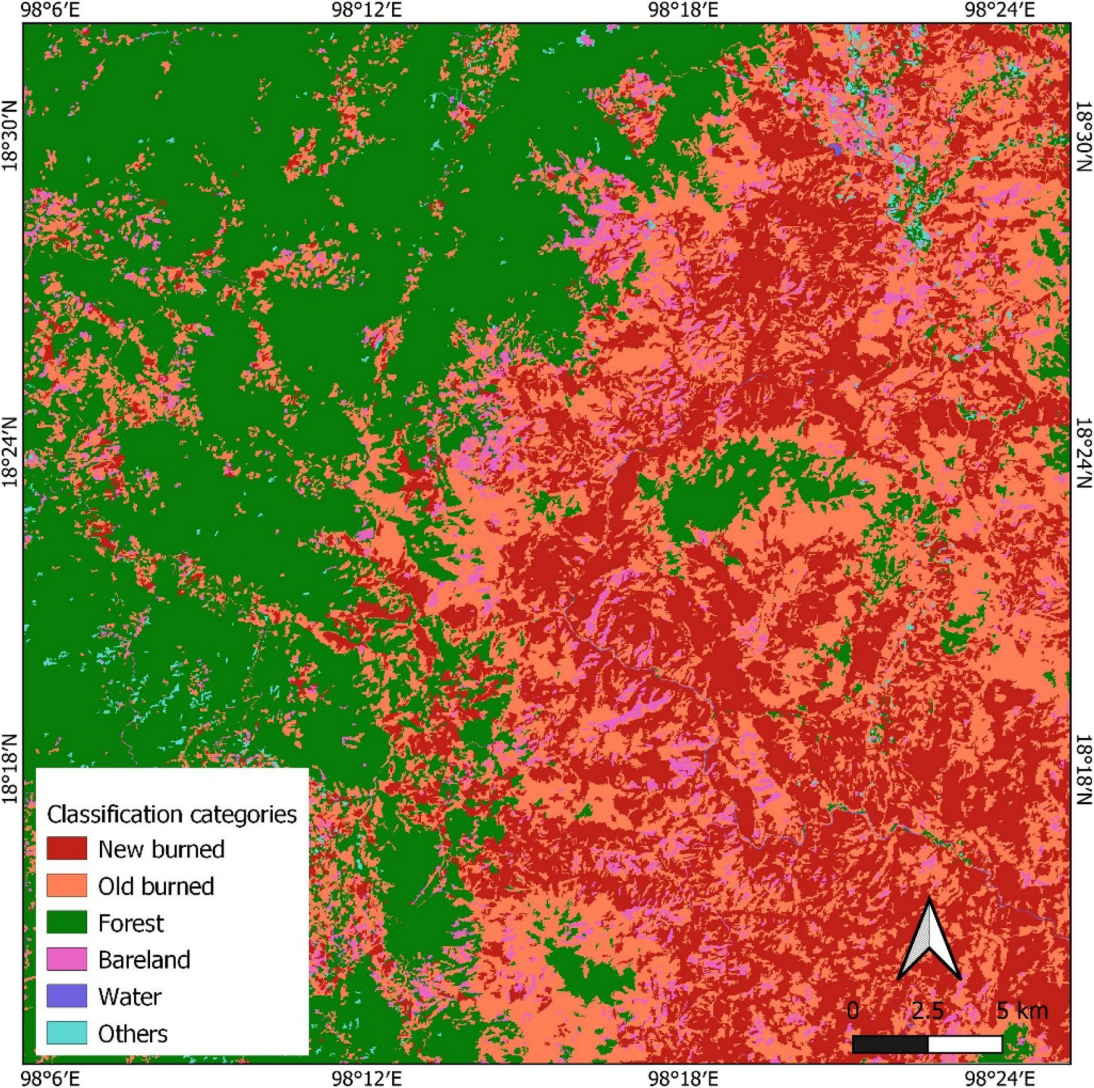
The final map of the burned area from OBIA classification. (This figure is the result of our analysis. The raw data was sourced from a public Sentinel-2 image available at https://scihub.copernicus.eu/. The segmentation analysis was conducted using Definiens eCognition version 10.3, which is available at https://geospatial.trimble.com/. The software was provided by the Department of Geoinformatics – Z_GIS, University of Salzburg, Austria. Further processing was done using QGIS version 3.26.2, available at https://qgis.org/en/site/).

The analysis presented in Fig. 6 provides a comprehensive overview of the land cover distribution within the study area. Notably, forests constituted the predominant land cover type, encompassing an impressive 52.25% of the total land area. Following closely behind was the new burn category, representing 24.30% of the land, while old burn areas accounted for 17.36%. Agriculture and other land cover types constituted 3.09% and 2.99% of the landscape, respectively, whereas water bodies were a mere 0.01%. This segmentation analysis strongly supports the effectiveness of the OBIA approach, as it successfully and accurately delineated burned areas. The total burned area within the study area, as extracted from Landsat 8 OLI imagery collected in January and February 2018 from the GISTDA dataset, amounted to 34.32 km^2^. Notably, the new burn category alone accounted for a substantial 100.94 km^2^ of the burned area, as illustrated in Figs. 6, 7 and 8. These findings underscore the significance of the new burn category in the context of burned area mapping.

Evaluating the effectiveness of any mapping method is contingent upon an assessment of its accuracy. This study employed a point accuracy assessment by comparing the OBIA burned area mapping results with the existing burned area dataset obtained from GISTDA. It is important to acknowledge that the two datasets did not align perfectly regarding acquisition dates, which may introduce variations. Nevertheless, the OBIA approach demonstrated an impressive 85.2% accuracy in identifying burned areas compared to 500 randomly selected burned points from the GISTDA dataset. These results emphasize that the synergy between OBIA and Sentinel-2A imagery, boasting a spatial resolution of 10 m, constitutes an efficient and promising methodology for accurately mapping burned areas in Thailand.

It is crucial to recognize that discrepancies between the burned areas identified in this study and the pre-existing burned area data could be attributed to various factors. These factors include disparities in spatial resolution, differences in image acquisition dates and times, and other contextual variables. For instance, the spatial resolution of Landsat imagery, set at 30 m, has faced criticism for its limited ability to accurately identify small-scale phenomena, such as selective logging. In this regard, imagery from the Sentinel-2 sensor, featuring a resampled spatial resolution of 10 m, may offer a more effective means of identifying and characterizing burned areas.

The attainment of an 85.2% accuracy rate in this study is a significant accomplishment, particularly given the complexity of the OBIA classification technique employed. OBIA stands out as a superior method compared to traditional pixel-based approaches, especially in the intricate task of identifying burned areas. This approach effectively discerns the subtle differences between burned and unburned areas by considering the spatial relationships and contextual information among groups of pixels. It is exceptionally beneficial in heterogeneous landscapes, where it reduces misclassification and generates more coherent maps, thus overcoming the common 'salt-and-pepper' effect seen in pixel-based analyses. Additionally, the ability of OBIA to integrate various data layers, including topographical and ecological information, enhances its accuracy, proving invaluable in environmental assessments where burn patterns are influenced by diverse geographical factors. Consequently, the high accuracy rate achieved in this study not only validates the methodological rigor but also highlights the effectiveness of OBIA in tackling complex landscape classification challenges.

However, this research identifies several limitations that merit attention for future study. A primary concern is the variation in spatial resolution and the timing of image capture, potentially leading to discrepancies between the identified burned areas and pre-existing datasets. The detailed features captured in the 10 m spatial resolution of Sentinel-2 imagery may be missed in the coarser 30 m spatial resolution of Landsat, indicating a need for further refinement of the methodology. Enhancements could include the incorporation of more comprehensive data layers and advancements in image processing techniques. The study also underscores the need to apply the OBIA method across various environmental scenarios and land cover types, which could provide valuable insights into its versatility and effectiveness. Moreover, future research might explore the integration of sophisticated machine learning algorithms with OBIA, enhancing the method's adaptability and precision across different geographical terrains and under diverse conditions. Conducting comparative analyses of OBIA results with those obtained from higher-spatial resolution imagery or alternative satellite data sources is another recommended avenue for future research. Such comparative studies would be instrumental in assessing the consistency and reliability of OBIA outcomes. Addressing these areas is critical for the progression of remote sensing and its application in environmental monitoring. In conclusion, this study accentuates the necessity for ongoing development and refinement of remote sensing methods to improve environmental analysis and management tools.

## Conclusion

To summarize, this study effectively endorses a streamlined method for detecting burned areas, leveraging four key Sentinel-2 imagery bands—Band 2 (Blue), Band 3 (Green), Band 4 (Red), and Band 8 (NIR), in tandem with NDVI, at a 10 m spatial resolution. This technique has shown remarkable success, achieving an 85.2% accuracy in identifying burned regions, thereby proving its merit in precise and efficient burn scar mapping. The use of OBIA with Sentinel-2 imagery further enhances its promise for rapid and accurate assessments, a vital aspect in emergency situations. This research echoes the findings of Polychronaki and Gitas^20^ in the efficacy of OBIA for burned area detection. It also aligns with the innovative approach of Kulinan et al.^21^, who integrated machine learning with Sentinel-2 imagery for wildfire damage assessment, presenting a different analytical perspective that could impact accuracy, processing time, and adaptability in diverse scenarios.

Moreover, this study's application of OBIA to Sentinel-2 imagery dovetails with prior research underscoring its success in accurately detecting burned areas in specific pilot areas. While acknowledging the potential for variations in different contexts, the research underscores the need for methodological adaptation to augment the growing body of knowledge in this field. Its significance extends beyond just Chiang Mai, addressing common issues in regions affected by agricultural burning and forest fires, like Southeast Asia.

The study underscores the efficacy of a simple yet effective approach, utilizing the sophisticated features of Sentinel-2 imagery and OBIA, to notably improve remote sensing capabilities for environmental monitoring and management. This research is a valuable addition to the ongoing efforts in efficient and effective burned area detection, highlighting the utility and effectiveness of streamlined methods in environmental and disaster response contexts.

## Data Availability

The data that support the findings of this study are available from the corresponding author, Chainda Suwanprasit, upon reasonable request. The Sentinel-2 satellite imageries are available from https://scihub.copernicus.eu/.
